# A Non-Lethal Traumatic/Hemorrhagic Insult Strongly Modulates the Compartment-Specific PAI-1 Response in the Subsequent Polymicrobial Sepsis

**DOI:** 10.1371/journal.pone.0055467

**Published:** 2013-02-08

**Authors:** Pierre Raeven, Alma Salibasic, Susanne Drechsler, Katrin Maria Weixelbaumer, Mohammad Jafarmadar, Martijn van Griensven, Soheyl Bahrami, Marcin Filip Osuchowski

**Affiliations:** Ludwig Boltzmann Institute for Experimental and Clinical Traumatology in the Trauma Research Center of Allgemeine Unfallversicherungsanstalt, Vienna, Austria; University of Cincinnati, United States of America

## Abstract

**Introduction:**

Plasminogen activator inhibitor 1 (PAI-1) is a key factor in trauma- and sepsis-induced coagulopathy. We examined how trauma-hemorrhage (TH) modulates PAI-1 responses in subsequent cecal ligation and puncture (CLP)-induced sepsis, and the association of PAI-1 with septic outcomes.

**Methods:**

Mice underwent TH and CLP 48 h later in three separate experiments. In experiment 1, mice were sacrificed pre- and post-CLP to characterize the trajectory of PAI-1 in plasma (protein) and tissues (mRNA). Post-CLP dynamics in TH-CLP (this study) and CLP-Only mice (prior study) were then compared for modulatory effects of TH. In experiment 2, to relate PAI-1 changes to outcome, mice underwent TH-CLP and were sampled daily and followed for 14 days to compare non-survivors (DEAD) and survivors (SUR). In experiment 3, plasma and tissue PAI-1 expression were compared between mice predicted to die (P-DIE) and to live (P-LIVE).

**Results:**

In experiment 1, an early post-TH rise of circulating PAI-1 was contrasted by a delayed (post-TH) decrease of PAI-1 mRNA in organs. In the post-CLP phase, profiles of circulating PAI-1 were similar between TH-CLP and CLP-Only mice. Conversely, PAI-1 mRNA declined in the liver and heart of TH-CLP mice versus CLP-Only. In experiment 2, there were no DEAD/SUR differences in circulating PAI-1 prior to CLP. Post-CLP, circulating PAI-1 in DEAD was 2–4-fold higher than in SUR. PAI-1 increase heralded septic deaths up to 48 h prior but DEAD/SUR thrombomodulin (endothelial injury marker) levels were identical. In experiment 3, levels of circulating PAI-1 and its hepatic gene expression were higher in P-DIE versus P-LIVE mice and those increases closely correlated with liver dysfunction.

**Conclusions:**

Trauma modulated septic PAI-1 responses in a compartment-specific fashion. Only post-CLP increases in circulating PAI-1 predicted septic outcomes. In posttraumatic sepsis, pre-lethal release of PAI-1 was mostly of hepatic origin and was independent of endothelial injury.

## Introduction

Trauma [1] and sepsis [2], [3] are among the leading causes of death worldwide and up to 10% of sepsis cases are preceded by trauma [4]. In trauma patients without brain injury, acute and early deaths are typically due to hemorrhage, whereas sepsis is the third most frequent cause of late death (in 31% of cases), after multiple organ failure (40%) and acute lung injury (26%) [5], [6]. An increased severity of traumatic injury increases the risk of developing sepsis by 6 to 16-fold [7].

Both trauma and sepsis trigger the innate immune response, activate the endothelium and non-specific host defense mechanisms, and affect blood clotting homeostasis [8]–[10]. Although their mechanisms and evolution can be diverse (reviewed in [11] and [12]), trauma and sepsis alike cause a deregulation of the coagulation/fibrinolysis balance leading to coagulopathy of different severity [10], [13]–[15]. When present, coagulopathy increases mortality by approximately 4-fold in trauma [13] and by approximately 2-fold in sepsis [16]. A single traumatic challenge, depending on its severity, may independently affect the host response and facilitate development of sepsis. It has been demonstrated that rats were more sensitive to endotoxin following surgical trauma (*i.e.* implantation of intravascular catheters 1 to 7 days earlier) [17]. Depending on the mediator(s) studied, the more recent reports demonstrate that an initial traumatic and/or hemorrhagic injury may cause either “priming” or “desensitization” of the immune system [18], [19].

Plasminogen activator inhibitor type 1 (PAI-1), a member of the serine protease inhibitor family, is an antagonist of both tissue- and urokinase-plasminogen activators and is deemed to be the key inhibitor of fibrinolysis [20]. PAI-1 is expressed in various cell types, predominantly in endothelial cells and hepatocytes [20], [21]. Apart from its role in coagulopathy, PAI-1 has been also shown to be a relevant factor in basic defense processes such as leukocyte activation [22] and clearance of apoptotic neutrophils [23], in tissue remodeling, development of cancer, atherosclerosis and the metabolic syndrome (reviewed in [21]). PAI-1 can be found in an active form which spontaneously converts into a latent conformation (half life of less than 2 hours), or bound to vitronectin, its main stabilizing protein [21].

Studies in trauma and sepsis alone imply that PAI-1 plays a critical role in coagulopathy occurring after either of the above conditions [15], [24], [25]. Circulating levels of PAI-1 were lower immediately after trauma [11], [26], but higher at later time points post-injury [26], [27]. In sepsis, plasma levels of non-survivors were >2-fold higher compared to survivors [25], [28], [29] and patients with homozygosity for the 4G allele (PAI-1 4G/5G promoter polymorphism) were 2 to 3-fold more susceptible for development of septic shock and multiple organ dysfunction syndrome MODS, and had a 2-fold higher risk of dying of MODS compared to patients with different genotypes [30], [31]. Clinical studies demonstrated that sepsis alone induces a rapid increase of PAI-1 in the blood (37–39), while we and others have shown that the magnitude of the post-septic PAI-1 response is highly compartment-specific [25], [32]–[34]. Despite its key role in critical diseases, the evolution of PAI-1 in post-traumatic sepsis is virtually unknown. The only, to our knowledge, relevant experimental inflammatory 2-hit study reported a 2-fold increase of PAI-1 mRNA expression in the rat lung (other tissues/organs were not examined) after an intra-tracheal endotoxin administration following hemorrhage (compared to endotoxin after sham treatment) [35].

In the present study, we aimed to characterize the evolution of PAI-1 over the course of post-traumatic sepsis and to establish if and/or how the initial trauma-hemorrhage hit modulates the PAI-1 protein and gene expression responses in the subsequent polymicrobial sepsis. Specifically, we focused on PAI-1 gene expression and presence of the protein in different compartments (*i.e.* in the blood, major organs and vascular endothelium) and its association with early septic outcomes, endothelial injury and risk of death.

## Materials and Methods

### Animals

Four week old (n = 42, weight 20–25 g, experiment 1) and three-month old (n = 72, 30 g, experiment 2 and 3), female CD-1 outbred mice (Harlan, Italy) were used. Mice were kept in groups of five animals per Type-III cage on a 12 h light –dark cycle with temperature maintained between 22–24°C and provided with a standard rodent diet and water *ad libitum* throughout all experiments (see the *Ethics Statement* for approvals/ethical details). Cages were enriched with houses, wood wool for nesting as well as wooden boards, tunnels and small blocks for gnawing (Abedd Lab & Vet Service, Austria) to facilitate natural behaviour prior to and throughout the experiments.

### Ethics Statement

All animal procedures were approved by the Viennese (Austria) legislative committee (Animal Use Proposal Permission no: 000794/2009/13) and conducted according to National Institute of Health guidelines.

Due to the severe nature of the TH-CLP model additionally complicated by the two age groups, a particular focus was put on monitoring of animals in the study to minimize suffering within the borders of the experimental design. All mice enrolled in the study were kept in the small in-house animal facility of our institute to enable optimal monitoring: the overall health status was checked by trained professionals (*i.e.* DVMs and/or MDs) at least three times per day during non-moribund disease period and every 2–3 h whenever mice were in an acutely severe state (defined by, *e.g.* decreased activity, progressing hypothermia, rapid weight gain; complete symptom list in [36]). To further enhance monitoring capacity and ensure maximal data reproducibility, all TH-CLP experiments were conducted in small sets of animals.

Given that in experiment 2 the objective of our study was to compare levels of circulating mediators between non-surviving and surviving mice, the time point of septic deaths was the most critical endpoint. This precluded us to follow typical humane endpoints as they are inadequate and too imprecise in critical illness models such as sepsis, shock, trauma and burns [36]: sacrificing mice using the classical (and accepted) humane endpoints (e.g. temperature, weight loss) in any of those severe disease experiments may be premature and produce type I and/or II errors. For example, in the CLP model, a weight gain (but not its loss) is more predictive of impending death, while hypothermia of 30°C has a positive predictive value for death (within 24 h) of only 56% [36].

Therefore, as suggested by Nemzek *et al.* [36] we followed a more accurate, empirically established set of guidelines that offered, although a very narrow, yet a feasible window of opportunity for induction of death. This was possible due to the frequent monitoring and did not hinder the experimental setup of this study. Specifically, mice were killed only at the end of each experiment or upon signs of imminent death (*i.e.* inability to maintain upright position/ataxia/tremor and prolonged/deep hypothermia and/or agonal breathing) by using deep inhalation anesthesia (isoflurane, Forane®, Abott, Austria) followed by an overdose of barbiturate (thiopental, Thiopental Sandoz®, Austria).

### Study Design

**Figure 1:**
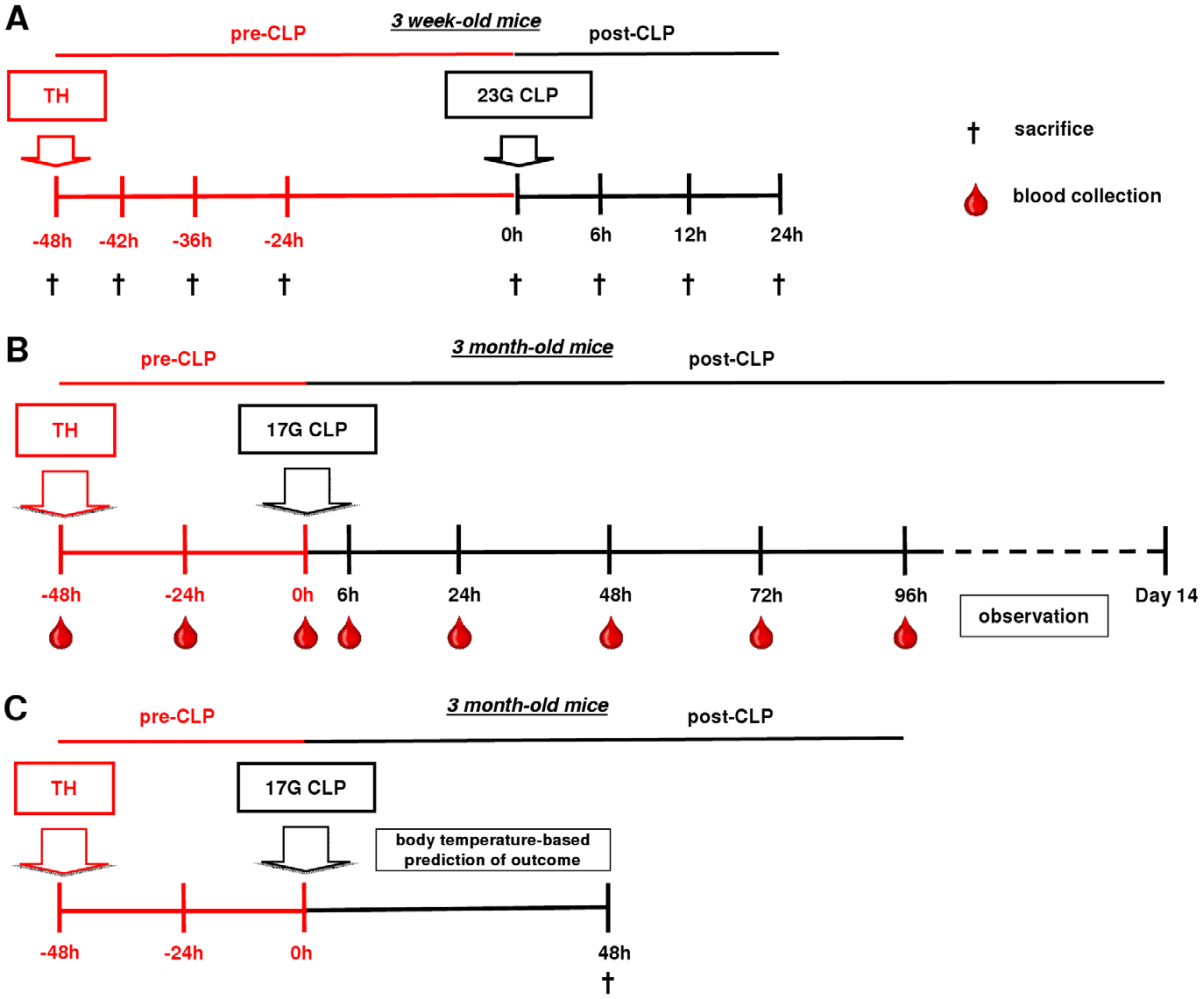
Schematic view of the experimental design of the two-hit models. A) 3 week-old mice were subjected to trauma and hemorrhage (TH) at −48 h followed by mild (23G) cecal ligation and puncture (CLP) sepsis at 0 h. The experimental period was divided into a pre-and post-CLP phase. At −48 , −42 h, −36 h, −24 h, 0 h, 6 h, 12 h and 24 h, mice (n = 6 per time point) were sacrificed (indicated by black crosses) and blood and organs were collected. B) 3 month-old mice (n = 42) were subjected to TH (−48 h) followed by medium-severity (17G) CLP-induced sepsis (0 h). The experimental period was divided into a pre-and post-CLP phase. From the TH time point (−48 h) on, 20 µl of blood was collected daily, and additionally at 6 h, until 96 h (day 6 post-TH/day 4 post-CLP). Animals were observed until death or day 14 post-CLP, whichever occurred first. C) 3 month-old mice (n = 30) were subjected to TH (−48 h) followed by CLP sepsis of medium-severity (17G) at 0 h. Pair of mice with a body temperature (BT) <28°C (predicted to die) and BT >36°C (predicted to live) were identified at the 48 h time-point and sacrificed.

The study consisted of three experimental parts displayed in Figure 1. In experiment 1, young female mice suffering from mild sepsis (as defined in the two-hit model subsection below) were sacrificed to obtain organ/tissue and blood samples for characterization of PAI-1 dynamics in different compartments (Fig. 1A). Four week old females were used to exclude possible estrogen-related variations and to reliably compare data from this study with the data from our previous study [25].

In experiment 2 (Fig. 1B), septic mice were never sacrificed but subjected to CLP of medium-severity, blood was sampled daily (see the *Blood sampling* subsection below) and followed until day 14 post-CLP. At the end of the study, mice were retrospectively divided into surviving (SUR) and dying (DEAD) subgroups. The 1100 ng/ml PAI-1 cut-off used for retrospective stratification of mice into DEAD and SUR was selected from the pooled values of circulating PAI-1 measured between 6 72 h post-CLP (this study). In experiment 2 and 3 (below), three month old mice were chosen as they appropriately recapitulate an adolescent population of trauma victims and are more suitable for daily monitoring (e.g. larger volume of daily blood samples).

In experiment 3, mice were again subjected to TH-CLP (CLP of medium-severity) and were stratified into either predicted to die (P-DIE) or predicted to live (P-LIVE) and sacrificed in pairs (1∶1 ratio) at 48 h post-CLP (Fig. 1C). Stratification was based on the post-CLP changes in body temperature (BT) as CLP mice dying in the acute phase of sepsis typically develop medium-to-severe hypothermia [37], [38]. Specifically, TH-CLP mice displaying BT<28°C were identified as P-DIE, while mice with BT>36°C were identified as P-LIVE. The predictive accuracy of BT stratification (Table S1) was established based on a large database that included BT measurements from experiment 2 mice and TH-CLP mice (all females) from another study [39]. BT-based stratification has a number of advantages: 1) P-DIE mice are fully symptomatic, thus are closer to death compared to IL-6-based prediction [40], 2) the BT separation is highly reliable (Table S1), and 3) maximal comparison consistency can be obtained by selecting any specific post-CLP day for sacrifice. In experiment 3, the 48 h was selected as an optimal time-point – a total of nine P-DIE/P-LIVE pairs were identified and sacrificed for comparative analysis.

### Two-hit Model

We used a post-traumatic sepsis model originally described by van Griensven *et al.* [41] with additional modifications for improved clinical relevance as detailed elsewhere [42]. All surgical procedures were performed in the morning, between 8–10 am.

#### Trauma/hemorrhage – the first hit

Briefly, mice were anesthetized (induction 3%, maintenance 1.5% isoflorane) and subjected to a non-comminuted, unilateral midshaft femur fracture with local tissue damage immediately followed by hemorrhage (TH, −48 h time-point). The femur fracture was produced by a brief compression with custom-designed blunt pliers. The effectiveness and reproducibility of injury was confirmed by X-ray. Hemorrhage was performed via retro-orbital puncture to produce a 40% blood loss of the total blood volume (calculated as 6% of the total body weight; individually in each mouse). Overall, the TH step was typically sub-lethal with only incidental deaths (0–5% mortality). All pre-CLP deaths in our model occurred within the first 2 h of TH – no late TH mortality was ever observed [42]. Post-TH, mice were subcutaneously resuscitated with 0.9% saline with four times the volume of shed blood: 1 mL containing analgesia (0.05 mg/kg buprenorphine, Buprenovet®) was given immediately after hemorrhage (simulating the phase of restricted resuscitation), while the remaining volume was administered 1 h post-TH (simulating the phase of unrestricted resuscitation).

#### Polymicrobial sepsis – the second hit

Mice were subjected to CLP (0 h time-point) at 48 h post-TH. We followed the original CLP protocol by Wichterman *et al.* [43] with relevant modifications [42]. Given that two different levels of CLP severity were employed in our study, we utilized two different needle gauges for puncturing the cecum: a 23-gauge (23G) needle was used in the first experiment (Fig. 1A), while a 17G needle in experiments 2 and 3 (Fig. 1B and C). As previously established, CLP with 23G needle ensures 100% survival within the first 24 post-surgery (and minimal long-term mortality), while 17G needle produces approx. 40% mortality at day 5 post-CLP (day 7 post-TH) [42]. Beginning at 0 h until the sacrifice time-point (experiment 1 and 3) or day 5 post-CLP (experiment 2), septic mice were resuscitated twice daily with 1 ml of saline including analgesia (0.05 mg/kg buprenorphine, Buprenovet®). Additionally, at 2 h after-CLP, each mouse was resuscitated by a single subcutaneous injection of 1 ml Ringer solution containing antibiotics (25 mg/kg imipenem/cilastatin, Zienam®, MSD, Austria) to emulate the clinical anti-microbial therapy [44].

We employed a subcutaneous instead of peritoneal route of administration given that the latter is more stressful to mice and it occasionally may result in inadvertent punctures of internal organs. Additionally, in CLP, intraperitoneal application is a potential confounder as progressing peritonitis may differentially modulate effects of antibiotic treatment among individuals. For pain management, all mice were administered metamizol (6 mg/kg, Novalgin®, Sanofi, Austria) via the drinking water from day 5 post-CLP until the end of each experiment. For all surgical procedures, mice were anesthetized with isoflurane (Forane®, Abbott, Austria). Given that post-CLP responses have been well described by us and others [25], [32]–[34], [45], sham surgeries were not performed to reduce the total number of mice in the study and to comply with the 3R tenet. All experiments were performed in small sets of animals at the time and the results of all separate experiments were combined in the final analysis.

### Blood Sampling

In experiment 1, 50 µl of blood was collected via facial vein puncture [46] immediately prior to sacrifice and at indicated time points (Fig. 1A). In experiment 2, mice were not sacrificed and 20 µl of blood was collected repetitively from each animal every 24 h (including an additional sample at 6 h post-CLP) [46] until day 5 (Fig. 1B). In experiment 3, 50 µl of blood was collected immediately prior to sacrifice via facial puncture (Fig. 1C). All blood samples were drawn into a pipette rinsed with ethylenediaminetetraacetic acid (EDTA) and then immediately diluted 1∶10 in phosphate-buffered saline containing 2% EDTA. After centrifugation (1000×g, 5 min, 22°C) supernatant (1∶10 diluted plasma) was removed and stored at –80°C until analysis.

### Complete Blood Count

The remaining cell pellets after centrifugation and removal of plasma were resuspended to the original 1∶10 diluted blood volume with CD3700 diluent (Abbot, Austria) containing EDTA. Complete blood counts (CBC) with differential were then performed on the CD3700 counter (Abbott, Austria) optimized for mouse blood.

### Organ and Metabolic Function Parameters

Alanine transaminase (ALT), aspartate transaminase (AST), blood urea nitrogen (urea), glucose, and lactate dehydrogenase (LDH) were analyzed in plasma samples using the Cobas c111 analyzer (Roche, Switzerland).

### Measurement of Circulating PAI-1 and Thrombomodulin

The detection of antigens in plasma samples was based on standard enzyme-linked immunosorbent assays (ELISA) using commercially available mouse kits for total PAI-1 (Innovative Research, USA) and soluble thrombomodulin (sTM, Uscn Life Science Inc., P.R. China) according to the manufacturer’s instructions. Compatibility of PAI-1 and sTM antigen assays with the EDTA-preserved blood (versus citrate-preserved) was previously validated [25]. To determine general PAI-1 dynamics in the systemic circulation, we assessed total (including the active, latent and bound forms) circulating PAI-1 rather than active PAI-1 alone.

### Gene Expression Analysis

At sacrifice (experiment 1), parts of the liver, kidney, lungs, heart and left cranial vena cava (LCVC) were collected, immediately snap-frozen in liquid nitrogen and stored at −80°C. In experiment 3, parts of the liver and LCVC were harvested and stored at −80°C. LCVC collections were pooled (n = 3–6 animals/group/time point for experiments 1 and 3) due to very small tissue volumes obtained from a single animal. Total RNA was extracted using the QIAzol Lysis Reagent/RNeasy Mini kit combination (Qiagen, Germany). RNA integrity was confirmed by agarose gel electrophoresis. Of the total RNA, 2 µg reverse transcribed into using anchored oligo-dT18-primers (Invitrogen, USA) and AMV Reverse Transcriptase (Finnzymes, Finland). Gene expression of murine PAI-1 and hypoxanthine-guanine phosphoribosyltransferase (HPRT) was analyzed by real-time polymerase chain reaction (PCR) on a CFX96 real-time cycler (Bio-Rad, Austria) using KAPA SYBR Green Universal 2X Mastermix (KAPA Biosystems, USA). Following primers were used: HPRT sense 5′-GCAAGTCTTTCAGTCCTGTCC-3′ and antisense 5′-GCAGCGTTTCTGAGCCAT-3′, PAI-1 sense 5′-TCCTCCACAGCCTTTGTCAT-3′ and antisense 5′-ACATCTCCACTTTCGTCCCA-3′. PCR protocol consisted of 3 min at 95°C for initial Taq-polymerase activation, followed by 40 cycles each including a denaturation step of 10 s at 95°C, an annealing step of 30 s at 60°C, and a 10 s extension step at 72°C with plate read. PAI-1 expression relative to HPRT (and normalized to 0 h time point) were calculated using the 2^−ΔCt^ method [47].

### Immunohistochemistry

At sacrifice (experiment 1, −48 h, −42 h and 24 h time points only), parts of the liver and lungs were collected, immediately snap-frozen in liquid nitrogen and stored at −80°C until further processing by immunohistochemistry (IHC). The tissue samples were embedded in OCT compound (Tissue-Tek, USA) and 5 µm-thick cryostat sections were cut. The sections were fixed in ice cold acetone and subsequently subjected to IHC staining. Endogenous peroxidase blocking was performed by treating the cryostat sections with 0.3% H_2_O_2_ in 80% methanol for 20 min. The sections were then washed with TRIS-buffered saline and incubated with anti-PAI-1 primary antibody (1∶500, Abcam, UK) for 1 h at room temperature. After another buffer rinse, all sections were incubated with an anti-rabbit polymer (Dako, Denmark) for 30 minutes at room temperature. After washing, staining was developed by using a peroxidase substrate kit (ImmPACT™ NovaRED™, Vector Laboratories, USA). The slides were then counterstained with hematoxylin, dehydrated and mounted permanently using an automatic glass coverslipper (CTM 6, Microm, Germany). Immunohistochemical controls were performed by replacing the primary antibody with antibody diluent (Zytomed Systems, Germany). Images were generated using the Olympus XC10 camera and OlyVIA dotSlide 2.4 software (Olympus, Austria).

### Statistical Analysis

Normality was tested in all recorded parameters and non-Gaussian data were log-transformed prior to further analyses. Normally distributed data were tested by the analysis of variance followed by the Tukey’s multiple comparison test, whereas skewed data were analyzed by the Mann–Whitney U-test. Data in tables that show comparisons of parameters collected in the TH-CLP (this study) and CLP-Only [25] mice, are presented as mean ± standard deviation. Data in graphs are presented on the original scale as box plot diagrams. Statistical assessment of LCVC expression in experiment 1 and 2 was not possible due to sample pooling.

All survival curves were plotted using the Kaplan-Meier method, and differences were analyzed by Log-rank (Mantel-Cox) test. The retrospective comparison of PAI-1 trajectory between SUR and DEAD (3∶1 ratio) data sets was performed at each time point separately using the unpaired t-test (normally distributed data) and Mann-Whitney test (skewed data). To characterize plasma PAI-1 using the time point of death as the reference point (regardless of the day of death), three consecutive pre-lethal PAI-1 values from each DEAD mouse were retrospectively tallied, displayed in an inverted fashion (i.e. 72 h, 48 h and 24 h prior to death) and matched against PAI-1 values from (randomly selected) SUR (alive at day 14) animals recorded on the same post-CLP day (1∶1 DEAD/SUR ratio).

Predictive accuracy of BT and circulating PAI-1 for post-TH-CLP outcomes was evaluated by the Receiver Operating Characteristics (ROC) curve and was expressed by the area under the curve (AUC). The predictive accuracy of the ROC-AUC was defined as: 0.9–1 = excellent, 0.8–0.9 = good, 0.7–0.8 = fair, 0.6–0.7 = poor and <0.6 = not useful. Correlations between selected parameters were tested by non-parametric analysis and expressed by Spearman’s rank correlation coefficient (ρ). All tests were carried out using Prism 5 (GraphPad, USA). The level of significance was determined at p<0.05.

## Results

### Trauma/Hemorrhage does not Alter the Dynamics of Circulating PAI-1 after the Subsequent CLP Hit

**Figure 2:**
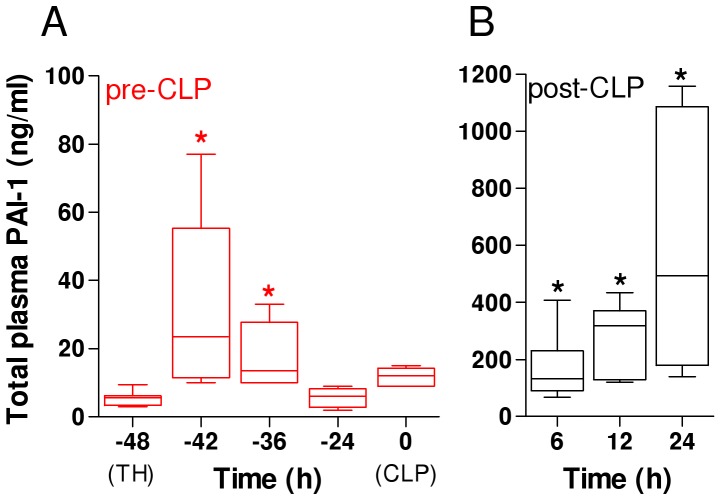
Circulating PAI-1 response to TH-CLP. 3 week-old mice were subjected to trauma and hemorrhage (TH) at −48 h followed by mild (23G) cecal ligation and puncture (CLP) sepsis at 0 h. At −48 h, −42 h, −36 h, −24 h, 0 h, 6 h, 12 h and 24 h, mice (n = 6 per time point) were sacrificed and blood was collected and analyzed for total plasminogen activator inhibitor type 1 (PAI-1) plasma concentration. Figure shows box plot diagrams, with whiskers indicating minimum and maximum and dots representing values outside of 1.5 inter-quartile range. *P<0.05 versus −48 h, § P<0.05 versus 6 h.

First, we portrayed the dynamics of circulating PAI-1 in the TH-CLP period (Fig. 2). At −48 h (baseline, immediately prior to TH), the total PAI-1 plasma concentration was 5.4 ng/ml (Fig. 2A). TH induced an elevation of plasma PAI-1 by 6-fold (32 ng/ml) at −42 h (6 h post-TH), followed by a decrease to the baseline level at −24 h (5.7 ng/ml). CLP (2^nd^ hit) provoked a gradual increase of circulating total PAI-1 from 6 h onward (32- and a 52-fold rise at 6 and 12 h) with the peak value (590 ng/ml) at 24 h (Fig. 2B). Pre-and post-CLP trajectories of selected hematological parameters (CBC) are shown in Table S2.

Second, we compared the evolution of total PAI-1 release into the blood after post-traumatic sepsis versus sepsis alone (Table 1). Baseline PAI-1 levels in mice subjected to TH-CLP (this study; measured at −48 h) and CLP-Only (previous study [25]) were identical (5.4 ng/ml). In TH-CLP mice, PAI-1 concentration at 0 h (immediately prior to CLP) was 50% higher compared to CLP-Only mice at 0 h (i.e. baseline) (Table 1). However, in both groups, PAI-1 measured post-CLP displayed an identical trajectory: its circulating concentrations rose gradually and were similar at all of the time points assessed.

**Table 1:** Circulating PAI-1 during the post-CLP phase in post-traumatic sepsis versus sepsis alone.

Plasma	0 h	6 h	12 h	24 h
TH-CLP	11.8±2.8*	168.5±121.9	278.5±126.0	590.0±432.8
CLP-Only§	5.4±2.1	97.1±35.7	422.3±323.6	1194±759.9
fold difference	2.2	1.7	0.7	0.5
P-value	0.008	0.096	0.427	0.934

Three week-old mice were subjected to trauma and hemorrhage (TH) followed by polymicrobial cecal ligation and puncture (CLP) sepsis or CLP alone (CLP-Only). At 0, 6, 12 and 24 h post-CLP, mice (n = 6 mice per time point) were sacrificed and blood was collected and analyzed for total plasminogen activator inhibitor type 1 (PAI-1) in plasma. Data as mean ± SD (TH-CLP and CLP-Only rows) representing 5–6 mice per time point. Fold PAI-1 difference between TH-CLP and CLP-Only.

§ Values obtained from a separate study (22).

* P<0.05 versus CLP-Only.

### Trauma/Hemorrhage Modulates the PAI-1 Gene Expression in the Post-CLP Phase

**Figure 3:**
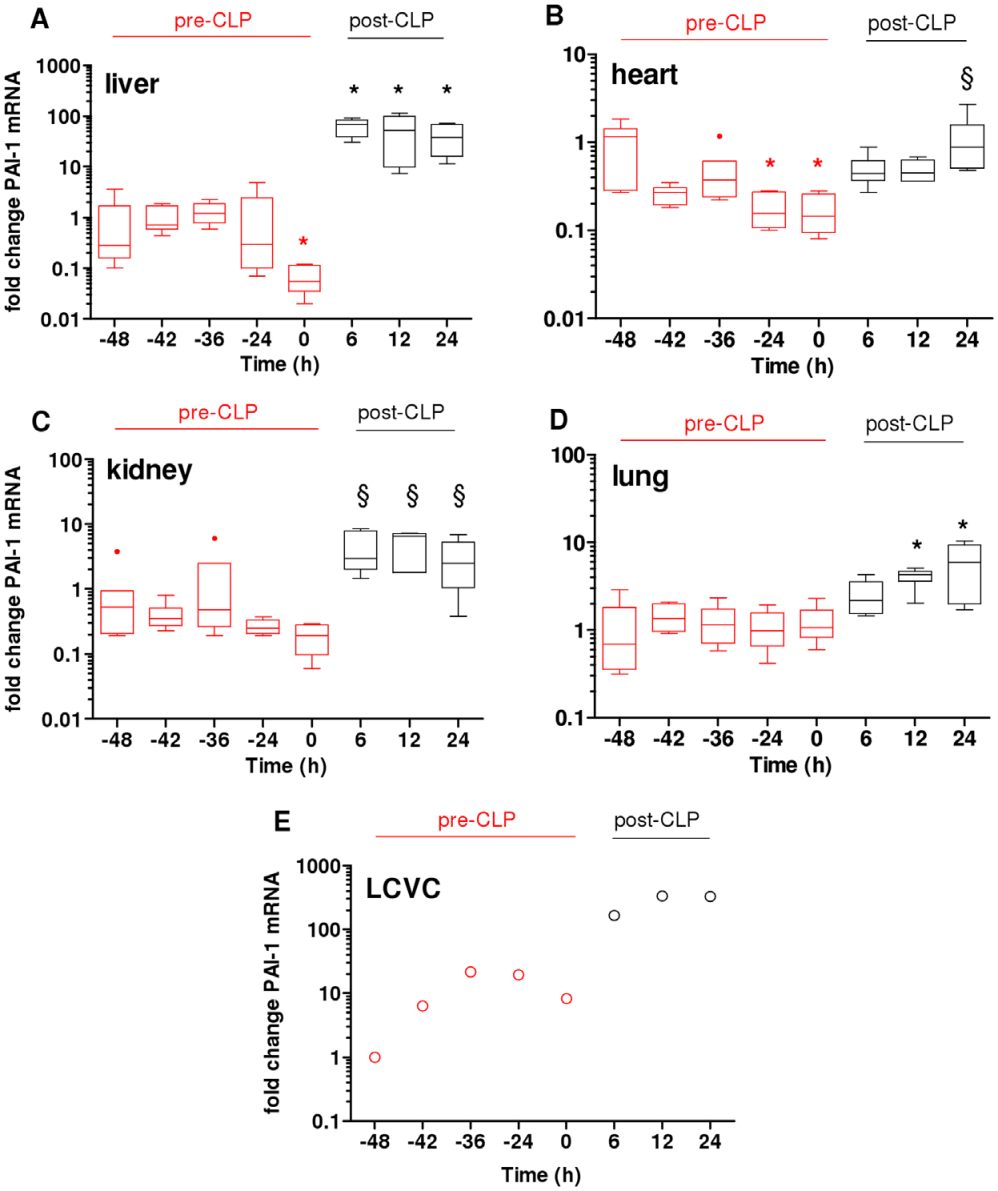
Organ-specific PAI-1 gene expression after TH-CLP. 3 week-old mice were subjected to trauma and hemorrhage (TH) at −48 h followed by mild (23G) cecal ligation and puncture (CLP) sepsis at 0 h. At −48 h, −42 h, −36 h, −24 h, 0 h, 6 h, 12 h and 24 h, mice (n = 6 per time point) were sacrificed and organs were collected and analyzed for expression of plasminogen activator inhibitor type 1 mRNA in the A) liver, B) heart, C) kidney, D) lung and E) left cranial vena cava (LCVC). Panels A-D show box plot diagrams representing 5–6 mice per time point, with whiskers indicating minimum and maximum and dots representing values outside of 1.5 inter-quartile range. In panel E, each LCVC dot represents a pooled collection of samples (n = 5–6 mice per time point). *P<0.05 versus −48 h; §P<0.05 versus 0 h.

In the next step, we characterized the trajectory of PAI-1 mRNA over the TH-CLP duration in various organs (Fig. 3). TH alone induced a strong PAI-1 mRNA decrease in the liver (by 93% at 0 h, Fig. 3A) and heart (by 83% at −24 h and 0 h, Fig. 3B). In the kidney and lungs, PAI-1 mRNA transcription was not affected by TH (Fig. 3 C–D). A statistically insignificant increasing trend (20-fold peak rise at −24 h) in PAI-1 mRNA was observed in the LCVC (Fig. 3E).

2^nd^ hit CLP induced an immediate (from 6 h onward, lasting up to 24 h) increase of PAI-1 mRNA the liver (>600-fold vs. 0 h; Fig. 3A) and a statistically insignificant rise (>20-fold vs. 0 h; Fig. 3E) in the LCVC. A milder increase of PAI-1 mRNA was recorded in the heart (3 to 7-fold; Fig. 3B) and kidney (Fig. 3C). In contrast, CLP did not consistently up regulate PAI-1 mRNA in the lungs, although PAI-1 mRNA level at 12 h was 4-fold (p<0.05) higher compared to baseline (Fig. 3D). Pre-and post-CLP trajectories of circulating ALT and AST as well as urea, glucose and LDH are shown in Figure S1.

Similar to the comparison in Table 1, we next compared the trajectory of PAI-1 mRNA fluctuations between post-traumatic sepsis and sepsis alone (i.e. the post-CLP phases in both groups, Table 2). At 0 h (i.e. immediately before CLP), PAI-1 gene expression in TH-CLP animals was lower in the liver (by −93%), kidney (−81%) and heart (−83%), but not in the lung compared to CLP-Only mice. In the post-CLP phase, PAI-1 mRNA level in TH-CLP mice was significantly lower in the liver (−83% at 24 h) and the heart (−78% at 12 h), but not in the kidney and lung compared to CLP-Only animals. PAI-1 mRNA in the LCVC appeared to increase faster after TH-CLP than CLP-Only but this comparison was not statistically verified due to sample pooling.

**Table 2:** Organ/tissue PAI-1 mRNA expression during the post-CLP in posttraumatic sepsis versus sepsis alone.

		0	6	12	24
Liver	TH-CLP	0.07±0.04*	46.53±23.92	55.63±48.01*	41.35±26.43*
	CLP-Only§	1.00±1.31	96.17±84.28	127.0±100.90	246.7±262.20
	fold difference	0.07	0.67	0.44	0.17
	P-value	0.0047	0.8149	0.1471	0.0103
Heart	TH-CLP	0.17±0.08*	0.49±0.21	0.48±0.13*	1.11±0.81
	CLP-Only§	1.00±0.61	1.19±0.73	2.17±1.81	0.92±1.17
	fold difference	0.17	0.42	0.22	1.21
	P-value	0.0043	0.1255	0.0168	0.2729
Kidney	TH-CLP	0.18±0.09*	4.52±3.08	5.13±2.62	3.01±2.35
	CLP-Only§	1.00±1.27	2.47±3.36	2.23±2.20	5.00±11.08
	fold difference	0.19	1.83	2.3	0.6
	P-value	0.035	0.065	0.0559	0.5362
Lung	TH-CLP	1.22±0.63	2.49±1.09	4.04±1.05	5.72±3.75
	CLP-Only§	1.00±1.06	2.34±1.08	2.78±1.66	3.66±3.68
	fold difference	1.21	1.07	1.45	1.56
	P-value	0.3095	0.9626	0.1807	0.2254
LCVC	TH-CLP	8.28	166.57	334.69	329.32
	CLP-Only§	1.00	4.30	14.29	140.90
	fold difference	8.28	38.74	23.42	2.34
	P-value	N/A	N/A	N/A	N/A

3 week-old mice were subjected to trauma and hemorrhage (TH) followed by polymicrobial cecal ligation and puncture (CLP) sepsis or CLP alone (CLP-Only). At 0, 6, 12 and 24 h post-CLP, mice (n = 6 mice per time point) were sacrificed and organs were collected and analyzed for total plasminogen activator inhibitor type 1 (PAI-1) mRNA expression. Data as mean ± SD (TH-CLP and CLP-Only rows) representing 5–6 mice per time point. Fold PAI-1 difference between TH-CLP and CLP-Only.

§ Values obtained from a separate study (22).

* P<0.05 versus CLP-Only. N/A, not applicable.

**Figure 4:**
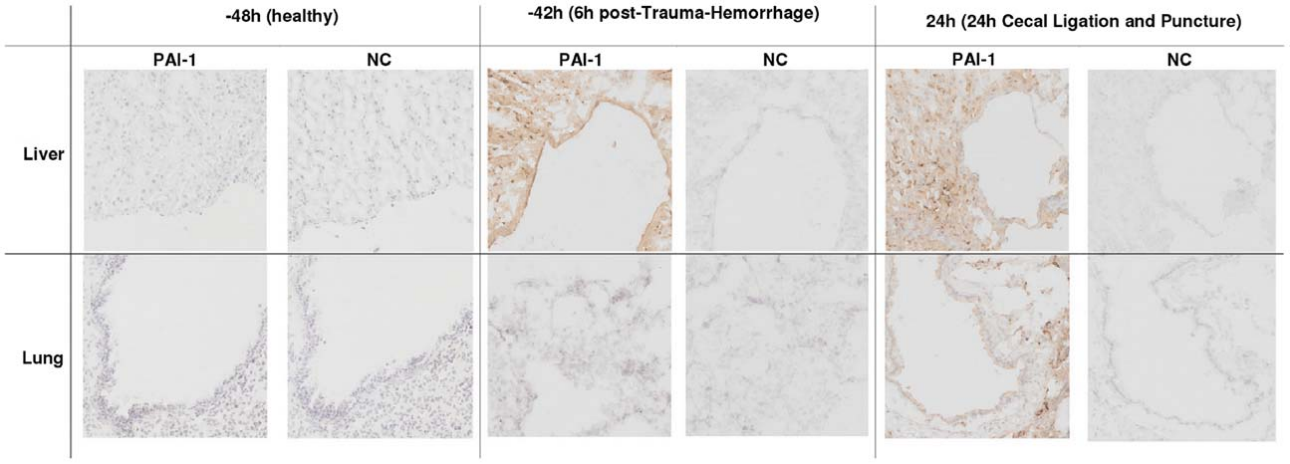
Immunohistochemical staining of PAI-1 in the liver and lung post-TH and TH-CLP. 3 week-old mice were subjected to trauma and hemorrhage (TH) at −48 h followed by mild (23G) cecal ligation and puncture (CLP) sepsis at 0 h. At −48 h, −42 h and 24 h, mice were sacrificed and organs were collected and stained for plasminogen activator inhibitor type 1 (PAI-1) in the liver and lung. Representative images for 3–6 animals per time point per tissue are shown. NC, negative control for PAI-1 (incubated without primary antibody). Original magnification x100.

To corroborate the above changes, we additionally examined the expression of PAI-1 protein in the liver and lungs by IHC (Fig. 4). At baseline, there was no evident expression of PAI-1. Whereas both TH (1^st^ hit) and CLP (2^nd^ hit) increased PAI-1 protein expression in the liver (at −42 h and 24 h), only post-traumatic CLP (but not TH alone) triggered a moderate expression of PAI-1 protein in the lungs.

### Increase of Circulating PAI-1 in Post-traumatic Sepsis is Associated with Worse Outcome

In the second part of the study, we investigated the relationship between PAI-1 in plasma and TH-CLP outcome. Mice subjected to TH-CLP were never sacrificed but sampled daily (until day 5 post-CLP). At the end of the study, all animals were retrospectively divided into either dying (DEAD) or surviving (SUR) and compared (see Materials and Methods for details).

**Figure 5:**
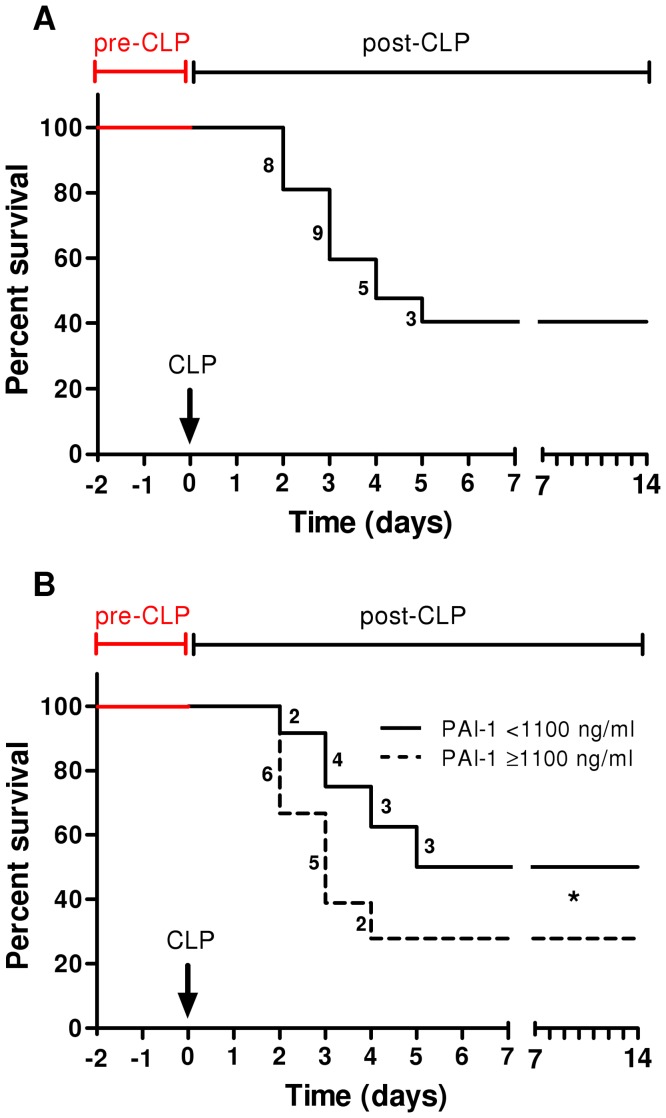
Increased plasma PAI-1 at 24 h post-TH-CLP is associated with lower 14-day survival. 3 month-old mice (n = 42) were subjected to trauma and hemorrhage (TH) at −48 h followed by mild (23G) cecal ligation and puncture (CLP) sepsis at 0 h. From the TH time point on, 20 µl of blood was collected daily and additionally at 6 h, until 96 h (day 6 post-TH/day 4 post-CLP). Animals were observed until death or day 14 post-CLP, whichever occurred first. A) 14-day survival. B) Survival of mice based on plasma PAI-1 at 24 h post-CLP below or above a subjective threshold. Digits on the graph indicate the number of deaths at the indicated time point. *p<0.05 versus <1100 ng/ml. CLP time point indicated by arrow.

TH-CLP mortality reached 60% by day 14 post-CLP (Fig. 5A). To test whether an early raise in PAI-I was associated with an increased mortality, survival curves were re-plotted to divide mice based on their circulating PAI-1 (cut-off set at 1100 ng/ml) measured at 24 h post-CLP (Fig. 5B). TH-CLP animals with plasma PAI-1 level ≥1100 ng/ml showed 22% higher mortality compared to their counterparts with PAI-1<1100 ng/ml (n = 24; p<0.05).

### Circulating PAI-1 Predicts Death after but not Prior to the CLP Hit

**Figure 6:**
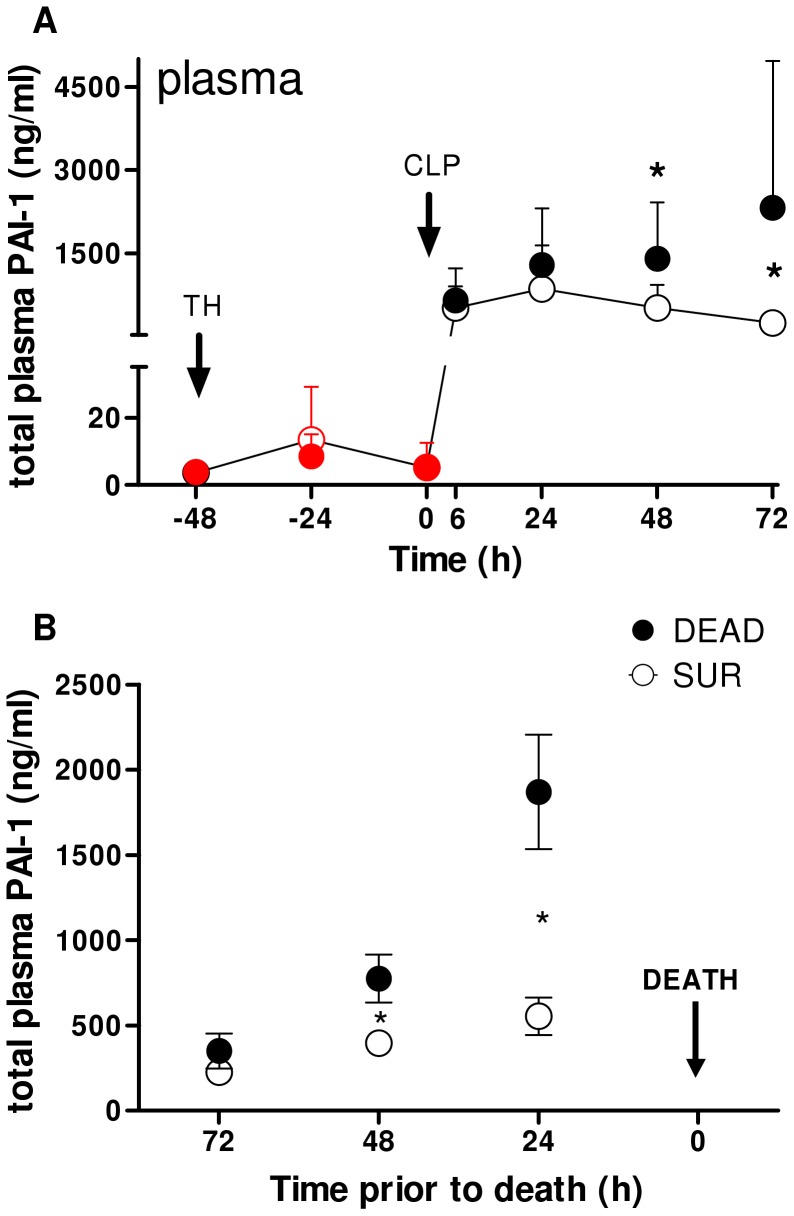
Plasma PAI-1 is increased in dying versus surviving mice. 3 month-old mice (n = 42) were subjected to trauma and hemorrhage (TH) at −48 h followed by mild (23G) cecal ligation and puncture (CLP) sepsis at 0 h. From the TH time point on, 20 µl of blood was collected daily and additionally at 6 h, until 96 h (day 6 post-TH/day 4 post-CLP. Animals were observed until death or day 14 post-CLP, whichever occurred first. The experimental period was divided into a pre-and post-CLP (red and black dots, respectively) phase. A) Time course of total circulating plasminogen activator inhibitor type 1 (PAI-1) of dying (DEAD, filled dots) and surviving (SUR, empty dots) mice. For SUR, n = 17 at each time point. For DEAD: n = 24 at −48 h, −24 h, 0 h and 6 h; n = 23 at 24 h; n = 15 at 48 h; n = 6 at 72 h. B) Circulating levels of PAI-1 of DIE (n = 24 at each time point) and time-matched levels of SUR (n = 24 at each time point) at the indicated time points prior to death. *p<0.05 versus SUR. CLP time point indicated by arrow.

Next, we compared the time course of circulating PAI-1 between SUR and DEAD mice (Fig. 6A). In the pre-CLP phase, plasma PAI-1 increase between DEAD/SUR mice was identical. In the post-CLP phase, however, plasma PAI-1 trajectories gradually separated over time. Compared to SUR, PAI-1 in DEAD was insignificantly elevated at 6 and 24 h (by 50 and 70%), approx. 2-fold higher at 48 h, and 4-fold higher at 72 h. This observation was corroborated by ROC analysis: the predictive accuracy for early death was poor at 6 h and 24 h (AUC = 0.57 and 0.65), good (AUC = 0.81) at 48 h and excellent (AUC = 0.9) at 72 h time point (Table S3).

Given that the exact time of the onset of sepsis is frequently unknown, we additionally assessed the exact time line of a feasible outcome-dependent separation. DEAD and SUR values were re-plotted in an inverted fashion using the day of death (days 1–5 post-CLP) as the reference point (Fig. 6B, see Materials and Methods). Prediction of post-CLP deaths was not possible earlier than 48 h prior to the event: while circulating PAI-1 levels in DEAD and SUR mice overlapped at 72 h before death, PAI-1 in DEAD (vs. SUR) gradually increased to reach a 2-fold and 3-fold difference at 48 h and 24 h prior to death, respectively.

### Pre-lethal Release of PAI-1 during Post-traumatic Sepsis is Independent of Endothelial Injury

**Figure 7:**
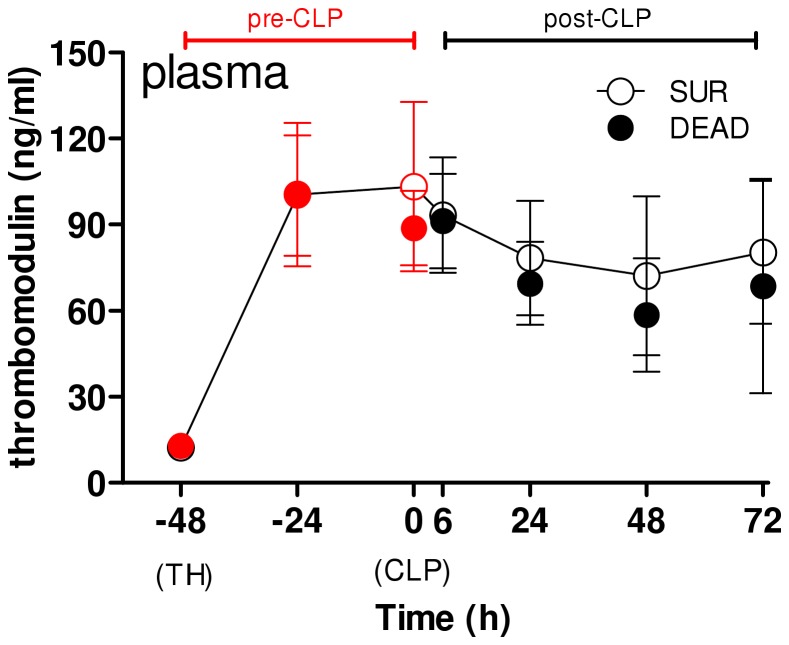
Plasma thrombomodulin level during TH-CLP develops irrespective of outcome. 3 month-old mice (n = 42) were subjected to trauma and hemorrhage (TH) at −48 h followed by mild (23G) cecal ligation and puncture (CLP) sepsis at 0 h. The experimental period was divided into a pre- and post-CLP (red and black dots, respectively) phase. From the TH time point (−48 h) on, 20 µl of blood was collected daily, and additionally at 6 h, until 96 h (day 6 post-TH/day 4 post-CLP). Animals were observed until death or day 14 post-CLP, whichever occurred first. For SUR, n = 17 for each time point. For DEAD: n = 24 at −48 h −24 h, 0 h and 6 h; n = 23 at 24 h; n = 15 at 48 h; n = 6 at 72 h. Figure depicts the time course of circulating thrombomodulin of dying (DEAD, black-filled dots) and surviving (SUR, white-filled dots) mice. CLP time point indicated by arrow.

To establish whether the above DEAD vs. SUR separation was reflected by the degree of endothelial injury, we studied the time course of sTM in the same set of mice (Fig. 7). The trajectory of sTM was identical between SUR and DEAD at all time points. Both groups had equal baseline values and TH induced an equivalent increase of sTM at −42 h (to approx. 100 ng/ml) that remained unchanged until 0 h (2^nd^ hit CLP). In the post-CLP phase, concentration of sTM was 5 to 7-fold higher than baseline but it was equal between SUR and DEAD at all time points.

### Pre-lethal Release of PAI-1 during Post-traumatic Sepsis Correlates with Exacerbated Hepatic PAI-1 Gene Expression and Liver Dysfunction

Finally, we aimed to establish the main source of pre-lethal PAI-1 release in post-traumatic sepsis. To achieve that, we employed a prediction of outcome based on changes in body temperature occurring during early CLP phase. This approach ensured high predictive accuracy (see Table S1 and Study Design) and at the same time, it allowed comparative organ analysis in mice with opposite sepsis severity.

**Figure 8:**
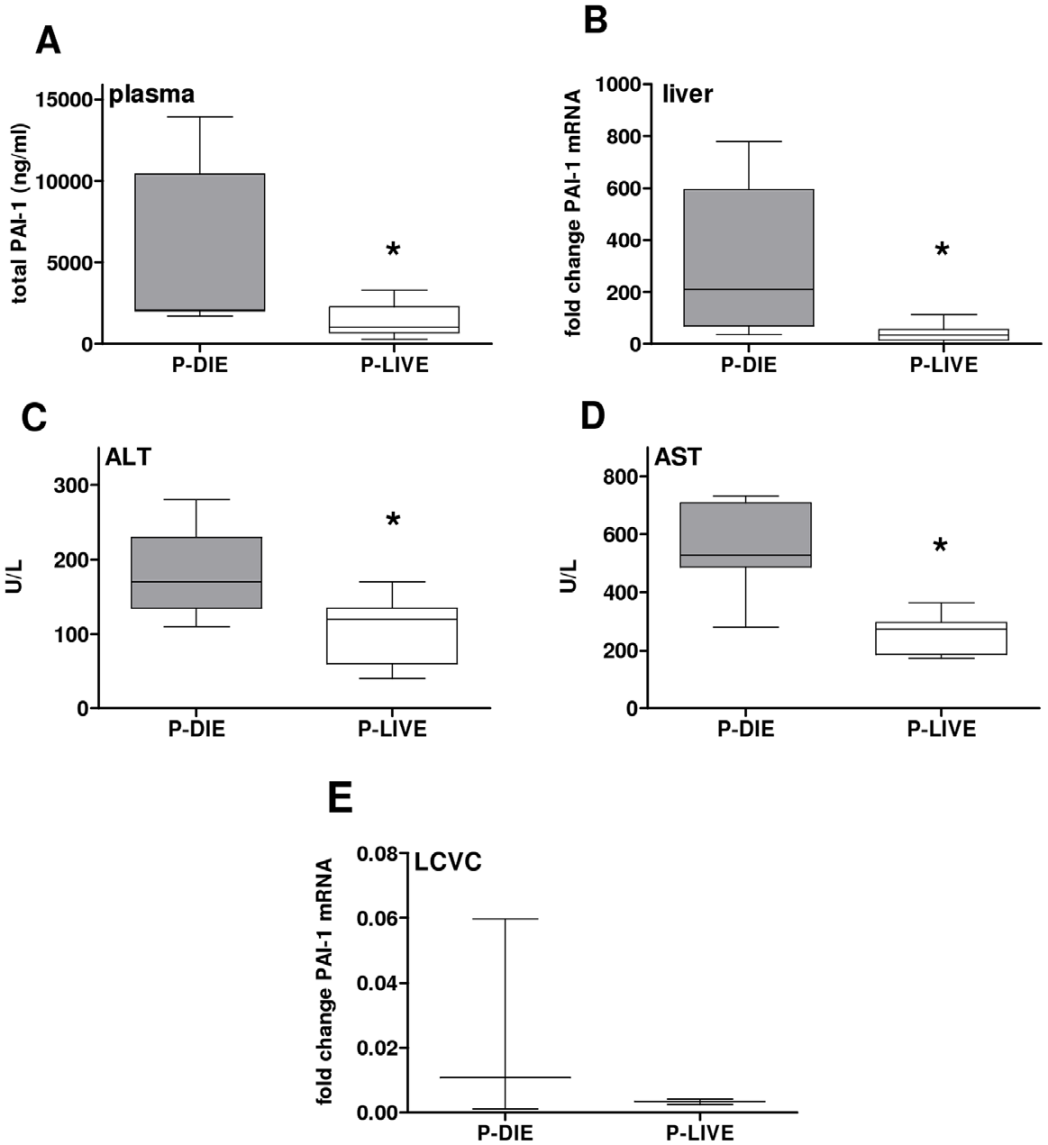
Release of PAI-1 into circulation is of hepatic origin and associated with liver dysfunction. 3 month-old mice (n = 30) were subjected to trauma and hemorrhage (TH) at −48 h followed by mild (23G) cecal ligation and puncture (CLP) sepsis at 0 h. Body temperature (BT) was measured in all mice in the post-CLP phase. Mice with body temperature (BT) <28°C (n = 9) were identified as predicted to die (P-DIE), while littermates with BT >36°C (n = 9) were identified as predicted to live (P-LIVE). Identified mice were instantly sacrificed (all at 48 h post-CLP) for collection of blood, liver and left cranial vena cava (LCVC). A) total plasminogen activator inhibitor type 1 (PAI-1) in plasma B) PAI-1 mRNA expression in the liver, C) plasma alanine transaminase (ALT), D) plasma aspartate transaminase (AST), E) PAI-1 mRNA expression in the left cranial vena cava (LCVC). Data as box plot diagrams representing 9 mice per column, with whiskers indicating minimum and maximum. In E, each column represents a total of 9 mice (3 pooled collections of LCVC samples, each collection comprising 3 mice). *P<0.05 versus <28°C.

Pairs of P-DIE and P-LIVE mice were identified and the concentration of circulating PAI-1 (and ALT, AST) and PAI-1 gene expression in the liver and LCVC were measured (Fig. 8). Compared to P-LIVE animals, a 2-fold increase of circulating PAI-1 in P-DIE mice (Fig. 8A) was accompanied by a 7-fold increase of PAI-1 mRNA in the liver (Fig. 8B). Notably, the above effect was paralleled by deterioration of liver function: compared to P-LIVE, there was an additional 2-fold increase in both ALT and AST in P-DIE mice (Fig. 8C and D). A strong correlation in outcome-dependent changes was recorded between circulating PAI-1 and all above-mentioned parameters (Table 3). In the LCVC, the level of PAI-1 mRNA expression was similar between P-DIE and P-LIVE mice (Fig. 8E).

**Table 3:** Correlation of circulating PAI-1 with its hepatic gene expression and liver function.

Variable 1	Variable 2	Spearman-ρ	95% CI	P-value	N
PAI-1plasma	PAI-1 mRNAliver	0.86	0.63 to 0.95	<0.01	18
PAI-1plasma	ALT	0.66	0.27 to 0.87	<0.01	18
PAI-1plasma	AST	0.66	0.26 to 0.87	<0.01	18

PAI-1, plasminogen activator inhibitor type 1; mRNA, messenger ribonucleic acid; ALT, alanine transaminase; AST, aspartate transaminase; CI, confidence interval; N, number of values.

3 month-old mice were subjected to trauma and hemorrhage (−48 h) followed by CLP sepsis of medium-severity (17G) at 0 h. In the post-CLP phase, mice with body temperature (BT) <28°C and littermates with BT >36°C were identified (1∶1 ratio) and instantly sacrificed. PAI-1 mRNA expression in the liver and plasma concentrations of total PAI-1, ALT and AST, and were correlated independent of BT and expressed by Spearman’s rank correlation coefficient (ρ).

## Discussion

A traumatic challenge may independently affect the evolution of the host response and regulation of important mediators during the development of a subsequent sepsis. In the present study, we investigated how initial trauma-hemorrhage influences the PAI-1 response in a following sepsis in mice, focusing on its expression in different compartments and its correlation with outcome.

An insight into the systemic compartment is clinically most important due to the potential diagnostic and/or predictive utility of measured parameters. The combination of the two current experiments (Fig. 1) with the data from our recently published CLP-Only study [25] allowed us to: 1) follow concentration of PAI-1 in the blood over the seven-day period (from the TH onset), 2) provide a comprehensive outcome-dependent characterization of early and delayed fluctuations of PAI-1 as mice transitioned from the trauma/hemorrhage to post-traumatic sepsis, and finally 3) establish the modulatory importance of the TH hit in post-traumatic CLP sepsis.

TH induced a marked increase of plasma PAI-1 (and a simultaneous decrease of hemoglobin, platelets and red blood cell count) that was followed by a complete recovery of PAI-1 (and of platelet counts) by the time of sepsis onset (Fig. 2A, Table S2). Thus, in the experimental context, this model [39], [41], [42], [48], [49] allows enough time for the TH-induced coagulation-fibrinolysis imbalance to be largely restored prior to the CLP hit, while, as we have recently demonstrated, the immuno-inflammatory system in TH mice remains highly activated at 0 h [39]. The above dynamics closely emulate the clinical scenario as the SIRS/inflammatory response in trauma patients can remain elevated for days [50], especially in patients developing subsequent secondary complications [51], [52], while the circulating PAI-1 subsides relatively quickly [27]. Moreover, two studies demonstrated that an acute blood loss in the volume comparable to the one shed in the current study (*i.e.* ∼40% of the total) leads to a period of severe hypotension as observed in patients suffering from hemorrhagic traumatic shock [53], [54]. Hence, this model provides an ideal milieu for investigating modulatory effects of the (TH - induced) isolated immuno-inflammatory component upon desired sequelae of secondary sepsis.

Regarding the PAI-1 gene/protein dynamics, this two-hit study reveals two novel findings. First, we demonstrate that the increase of circulating PAI-1 after TH hit did not evidently influence its subsequent dynamic after sepsis (Table 1). In general, both the post-CLP trajectory and the magnitude of the observed PAI-1 increase remained similar between the TH-CLP and CLP-Only groups. Although we did not measure it earlier than 6 h post-TH, the transient PAI-1 increase (at 6 and 12 hours after TH) is also reported in patients [27] and it likely reflects a compensatory rebound after the steep decrease of PAI-1 immediately after trauma [26]. In contrast, the increase of plasma PAI-1 during sepsis is expected to be a direct counter-regulatory response against induction of plasminogen activators and successive plasmin production. The post-CLP increase in PAI-1 expression is also triggered by systemic release of TNFα and IL-1β [12].

Second, we show a differential (TH-dependent) priming of the PAI-1 gene response in various organs and an evident mismatch between the mRNA expression and circulating PAI-1 protein. The liver has been identified as one of the most prominent sources of PAI-1 after both hemorrhage [55], [56] and CLP-induced sepsis [25], [33]. After TH alone, we observed an early trend to increasing hepatic PAI-1 gene expression (Fig. 3A) that was similar to the early hepatic PAI-1 mRNA increase reported by Yamashita *et al.* in hemorrhagic rats [55]. The delayed drop of PAI-1 mRNA in the liver and heart at 0 h (i.e. 48 h after TH) is currently unsubstantiated as this finding has not been scrutinized by others. In the acute phase of post-traumatic sepsis, we recorded a distinct upregulation of PAI-1 gene expression in the liver (also a strong trend to increases in the LCVC). Yet, the mRNA upregulation after two hits was much lower when compared to the hepatic PAI-1 expression recorded in CLP-Only mice (Table 2). Interestingly, while this elevation was remarkably constant across all time-points in TH-CLP mice, it increased additionally in CLP-Only animals. Regarding other organs, both heart and kidney remained quiescent whereas only a delayed, low-grade increase of PAI-1 mRNA was observed in the lung. In contrast to the aforementioned findings of Fan *et al.* (30), the increase of PAI-1 mRNA in the lung was not additionally modulated by TH (vs. CLP-Only, Table 2). This disparity can be explained by the difference in the magnitude/localization of the inflammatory response – a systemic one after CLP (this study) vs. an isolated response after a local (intratracheal) endotoxin administration (30). In addition, we have recently shown that acute CLP does not produce a significant lung injury in female CD-1 (ICR) mice [57]. Overall, the current data convincingly implicate liver as the chief source of PAI-1 production and release in post-traumatic sepsis.

Both clinical and experimental studies have shown that high levels of circulating PAI-1 early after the sepsis onset are associated with worse long-term outcome [25], [28], [58]. Pralong *et al.* found that 71% of the patients with PAI-1 levels of more than 550 ng/ml at time of entry to the study died within 7 days (acute septic deaths) compared to only 6% of patients with PAI-1 levels lower than 550 ng/ml [58]. We now report an identical relationship in post-traumatic sepsis, albeit at a different PAI-1 cut-off. Out of all mice with PAI-1 level higher than or equal to 1100 ng/ml (measured at 24 h post-CLP), 72% died within 7 days, compared to 50% of mice with PAI-1 below 1100 ng/ml (Fig. 5B). The outcome discrepancy between the two low-risk groups is largely due to the relatively dense accumulation of deaths within the first 5 days post-CLP in our 2-hit model compared to a more protracted mortality in the clinical study [58]. In patients dying of sepsis, plasma PAI-1 typically rises over time and remains high until the time of death, whereas PAI-1 in survivors peaks early and recovers to normal levels within approx. one week [24], [28]. We observed a similar trajectory; at 48 h and 72 h after the 2^nd^ hit (but not earlier) circulating PAI-1 in dying septic mice continued to rise, whereas it remained stable after the initial post-CLP increase in all survivors (Fig. 5A). Disappointingly, the trauma-induced fluctuations of PAI-1 during the post-TH phase (but pre-CLP), did not discriminate post-traumatic sepsis outcomes prior to its onset. In the context of a prospective risk-assessment in any patient suffering from post-traumatic sepsis, the impending outcome is a much more relevant reference point than the time of the onset of sepsis as the latter event may be easily overlooked. After re-plotting the data using the day of death as the reference time point (Fig. 6B), it became apparent that PAI-1 concentration in dying mice was 2 to 3-fold higher than in matched survivors, yet not earlier than 48 h prior to death. Although this time-window is identical to the effective time-span in predictive capacity of IL-6 (and other cytokines) for septic deaths [39], [45], [59], a relatively loose clustering of individual, outcome-based PAI-1 values disqualifies it as a potential stand-alone predictor of outcome in sepsis syndromes. Yet, when viewed collectively, the above findings invite a much more important speculation: i.e. that a detectable pre-lethal shift in the systemic coagulation/fibrinolytic (as well as inflammatory) responses occurs in a relatively advanced stage of sepsis (and irrespective of its chronological scale). In other words, given that septic deaths are not typically heralded immediately after the onset of the disease, it is suggestive that interventions undertaken even in late sepsis may effectively save lives – provided that they are appropriately fine-tuned.

Given that the vascular bed constitutes a rich source of PAI-1, the last investigative element of experiment 2 aimed to assess the relationship between the pre-lethal increase of circulating PAI-1 and the level of endothelial injury. Soluble thrombomodulin is regarded as an accurate marker of endothelial injury and both trauma and sepsis trigger its rapid release into the bloodstream [60], [61]. In our study, sTM increased immediately after TH and remained strongly elevated until day 3 post-CLP. This is indicative of a lasting and relatively high-degree of endothelial injury with the first hit serving as the main injurious trigger (given that 2nd hit did not further aggravate the magnitude of the TH-induced damage). Surprisingly, the strong outcome-dependent separation of circulating PAI-1 (Fig. 5) was not reflected by fluctuation of sTM: its trajectories in dying and surviving TH-CLP mice were nearly superimposable (Fig. 6). Given a relatively short half-life (<2 h) of PAI-1 and sTM in circulation [20], [61], the above data strongly imply that in post-traumatic sepsis, the pre-lethal PAI-1 release is independent of the level of endothelial injury.

Out of all investigated organs in our two-hit model, liver demonstrated the most robust and consistent expression of the PAI-1 gene. Therefore, in the final step of our study, we sought to characterize the outcome-dependent transcription of hepatic PAI-1 and correlate it with the release of circulating PAI-1 protein. Given that analyses of outcome-based gene expression in tissues were not possible in experiment 2, we devised an alternative protocol to enable accurate comparison of lethal versus non-lethal PAI-1 responses (see Study Design). Specifically, we relied on changes in body temperature to stratify TH-CLP mice into those predicted to die (P-DIE; hypothermic) and those predicted to live (P-LIVE; normothermic). The comparison revealed a strong outcome-dependent activation of PAI-1 transcription in the liver (but not in LCVC). Notably, circulating PAI-1 tightly correlated with its hepatic gene expression (Table 3) and pre-lethal increases in both parameters demonstrated a strong positive correlation with the level of liver dysfunction (by ALT and AST). While this does not establish a clear causal relationship, Lagoa *et al.*, suggested that hepatic expression of PAI-1 might directly influence liver function or *vice versa*. Using a rat model of hemorrhagic shock, they showed that generalized liver dysfunction as well as localized damage of hepatic endothelium were absent in PAI-1 knockout mice but this tissue preservation was gone after administration of stable PAI-1 [56]. Interestingly, they also observed a post-resuscitation shift of the immunohistochemical PAI-1 protein signal from hepatocytes into the endothelium-rich hepatic sinusoids. It remains to be verified whether similar effects can be triggered by systemic infections. However, the combined results of our study convincingly imply that liver (either parenchymal cells and/or hepatic endothelium) is the key contributor behind the robust pre-lethal release of circulating PAI-1 in post-traumatic sepsis.

In conclusion, we postulate that initial blunt trauma coupled with sublethal hemorrhage does not alter the concentration of PAI-1 in the plasma during subsequent sepsis, yet they strongly modulate PAI-1 gene expression in selected organs. Moreover, plasma PAI-1 in post-traumatic sepsis is chiefly secreted in the liver and its synthesis and release is higher in dying subjects than in survivors. Interestingly, the latter phenomenon is irrespective of the level of damage to vascular endothelium. Finally, since the TH-CLP model closely mimics the evolution of PAI-1 observed in trauma and/or septic patients it may serve as a relevant platform for pre-clinical testing of potential PAI-1-modulating therapeutics.

## Supporting Information

Figure S1 **Organ function response to TH-CLP.** 3 week-old mice were subjected to trauma and hemorrhage (TH) at-48 h followed by mild (23G) polymicrobial cecal ligation and puncture (CLP) sepsis at 0 h. At −48 h, −42 h, −36 h, −24 h, 0 h, 6 h, 12 h and 24 h, mice (6 per time point) were sacrificed and blood was collected and analyzed for A) alanine transaminase (ALT), B) aspartate transaminase (AST), C) blood urea nitrogen (urea), D) glucose, and E) lactate dehydrogenase (LDH) plasma concentrations. Figure shows box plot diagrams, with whiskers indicating minimum and maximum and dots representing values outside of 1.5 inter-quartile range. *P<0.05 versus −48 h, §P<0.05 versus 0 h.
(TIF)

Table S1 **Prediction of TH-CLP outcomes based on body temperature.** BT, body temperature; AUC, area under the curve; CI, confidence interval, NPV, negative predictive value for outcome; PPV, positive predictive value for outcome. 3 month-old female mice (n = 57) were subjected to trauma and hemorrhage (TH, −48 h) followed by cecal ligation and puncture (CLP) sepsis of medium-severity (17G) at 0 h. Body temperature was recorded in all mice in the post-CLP phase (days 1–5 post-CLP). Table displays predictive accuracy (within next 48 h) for BT measurements taken at 48 h post-CLP only.
(DOC)

Table S2 **Hematological response to TH-CLP.** 3 week-old mice were subjected to trauma and hemorrhage (−48 h) followed by mild (23G) polymicrobial cecal ligation and puncture (CLP) sepsis at 0 h. At −48 h, −42 h, −36 h, −24 h, 0 h, 6 h, 12 h and 24 h, mice (6 per time point) were sacrificed and blood was collected and analyzed. RBC, red blood cell count; PLT, platelet count; Hb, hemoglobin; WBC, white blood cell count; NEU, neutrophil granulocyte, LYM, lymphocyte count. ^a^P<0.05 versus −48 h, ^b^P<0.05 versus −42 h, ^c^P<0.05 versus −36 h, ^d^P<0.05 versus −24 h, ^e^P<0.05 versus 0 h, ^f^P<0.05 versus 6 h, ^g^P<0.05 versus 24 h, 05 versus all other time points. Data as mean ± SD; n ≥5 per time point.
(DOC)

Table S3 **Predictive accuracy of plasma PAI-1 for death.** AUC, area under the curve; ROC, receiver operating characteristic; CI, confidence interval. *P<0.05. Plasma PAI-1 levels obtained were evaluated by the ROC curve to determine the predictive accuracy for the outcome as expressed by the AUC. The predictive accuracy of the ROC-AUC was defined as: 0.9–1 = excellent, 0.8–0.9 = good, 0.7–0.8 = fair, 0.6–0.7 = poor and <0.6 = not useful.
(DOC)
