# Supplementary figures and images for: A Non-Lethal Traumatic/Hemorrhagic Insult Strongly Modulates the Compartment-Specific PAI-1 Response in the Subsequent Polymicrobial Sepsis
Source: PLoS One. 2013 Feb 8;8(2):e55467. doi: 10.1371/journal.pone.0055467 (PMC3568129; doi:10.1371/journal.pone.0055467)

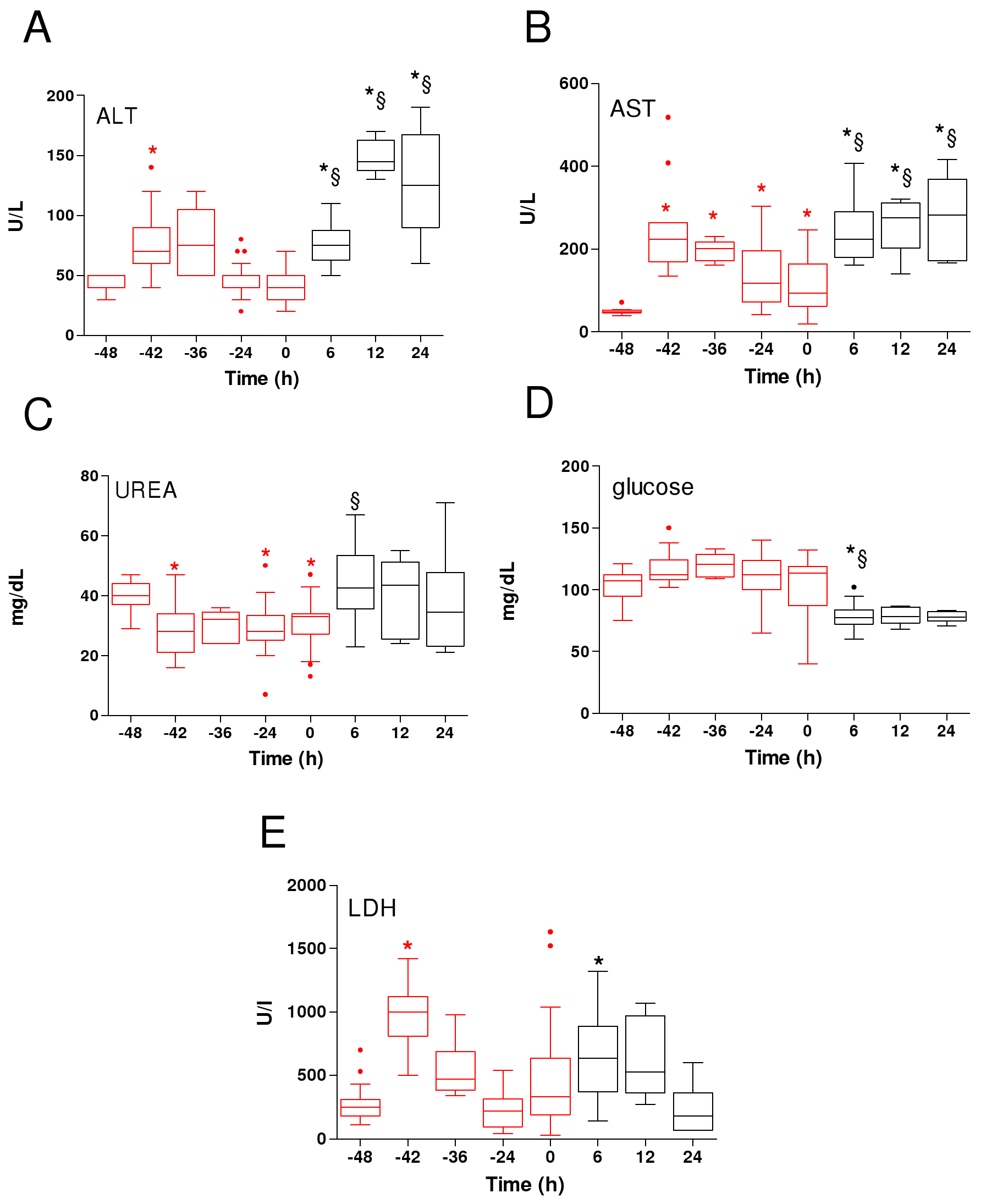

Supplement: Figure S1 — Organ function response to TH-CLP. 3 week-old mice were subjected to trauma and hemorrhage (TH) at-48 h followed by mild (23G) polymicrobial cecal ligation and puncture (CLP) sepsis at 0 h. At −48 h, −42 h, −36 h, −24 h, 0 h, 6 h, 12 h and 24 h, mice (6 per time point) were sacrificed and blood was collected and analyzed for A) alanine transaminase (ALT), B) aspartate transaminase (AST), C) blood urea nitrogen (urea), D) glucose, and E) lactate dehydrogenase (LDH) plasma concentrations. Figure shows box plot diagrams, with whiskers indicating minimum and maximum and dots representing values outside of 1.5 inter-quartile range. *P<0.05 versus −48 h, §P<0.05 versus 0 h. (TIF) [file pone.0055467.s001.tif]
