# Supplementary material for: A Non-Lethal Traumatic/Hemorrhagic Insult Strongly Modulates the Compartment-Specific PAI-1 Response in the Subsequent Polymicrobial Sepsis
Source: PLoS One. 2013 Feb 8;8(2):e55467. doi: 10.1371/journal.pone.0055467 (PMC3568129; doi:10.1371/journal.pone.0055467)
Supplement: Table S1 — Prediction of TH-CLP outcomes based on body temperature. BT, body temperature; AUC, area under the curve; CI, confidence interval, NPV, negative predictive value for outcome; PPV, positive predictive value for outcome. 3 month-old female mice (n = 57) were subjected to trauma and hemorrhage (TH, −48 h) followed by cecal ligation and puncture (CLP) sepsis of medium-severity (17G) at 0 h. Body temperature was recorded in all mice in the post-CLP phase (days 1–5 post-CLP). Table displays predictive accuracy (within next 48 h) for BT measurements taken at 48 h post-CLP only. (DOC) [file pone.0055467.s002.doc]

Table S1. Prediction of TH-CLP outcomes based on body temperature.

| **Predicted Outcome** | **BT** | **Specificity** | **Sensitivity** | **AUC** | **95% CI** | | **NPV** | **PPV** |
| --- | --- | --- | --- | --- | --- | --- | --- | --- |
| death | >28°C | 97% | 50% | 0.94 | 0.874 | 0.9991 | 90% | 81% |
| alive | <36°C | 100% | 72% | 0.94 | 0.874 | 0.9991 | 100% | 62% |

BT, body temperature; AUC, area under the curve; CI, confidence interval, NPV, negative predictive value for outcome; PPV, positive predictive value for outcome.

3 month-old female mice (n=57) were subjected to trauma and hemorrhage (TH, -48h) followed by cecal ligation and puncture (CLP) sepsis of medium-severity (17G) at 0h. Body temperature was recorded in all mice in the post-CLP phase (days 1-5 post-CLP). Table displays predictive accuracy (within next 48h) for BT measurements taken at 48h post-CLP only.
