# Supplementary material for: A Non-Lethal Traumatic/Hemorrhagic Insult Strongly Modulates the Compartment-Specific PAI-1 Response in the Subsequent Polymicrobial Sepsis
Source: PLoS One. 2013 Feb 8;8(2):e55467. doi: 10.1371/journal.pone.0055467 (PMC3568129; doi:10.1371/journal.pone.0055467)
Supplement: Table S2 — Hematological response to TH-CLP. 3 week-old mice were subjected to trauma and hemorrhage (−48 h) followed by mild (23G) polymicrobial cecal ligation and puncture (CLP) sepsis at 0 h. At −48 h, −42 h, −36 h, −24 h, 0 h, 6 h, 12 h and 24 h, mice (6 per time point) were sacrificed and blood was collected and analyzed. RBC, red blood cell count; PLT, platelet count; Hb, hemoglobin; WBC, white blood cell count; NEU, neutrophil granulocyte, LYM, lymphocyte count. aP<0.05 versus −48 h, bP<0.05 versus −42 h, cP<0.05 versus −36 h, dP<0.05 versus −24 h, eP<0.05 versus 0 h, fP<0.05 versus 6 h, gP<0.05 versus 24 h, 05 versus all other time points. Data as mean ± SD; n ≥5 per time point. (DOC) [file pone.0055467.s003.doc]

Table S2. Hematological response to TH-CLP.

| Time (h) | RBC (M/µl) | PLT (K/µl) | Hb (g/dl) | WBC (M/µl) | NEU (M/µl) | LYM (M/µl) |
| --- | --- | --- | --- | --- | --- | --- |
| -48 | 6.65 ± 0.76 | 1233 ± 228 | 12.38 ± 1.28 | 5.12 ± 2.3 | 0.77 ± 0.26 | 4.08 ± 2.15 |
| -42 | 4.20 ± 0.52^a^ | 929 ± 235 | 7.58 ± 0.79 ^a^ | 3.98 ± 1.49 | 1.17 ± 0.29 ^a^ | 2.69 ± 1.30 |
| -36 | 4.07 ± 0.53^a^ | 930 ± 166 | 7.43 ± 0.85 ^a^ | 3.45 ± 2.00 | 0.69 ± 0.48 ^b^ | 2.7 ± 1.84 |
| -24 | 4.50 ± 0.95^a^ | 960 ± 259 ^a^ | 8.39 ± 1.56 ^a^ | 6.2 ± 2.96 | 0.63 ± 0.24 ^b^ | 5.67 ± 3.17 |
| 0 | 4.61 ± 0.67^a^ | 1030 ± 392 | 8.97 ± 1.18 ^a^ | 7.27 ± 3.20 | 1.06 ± 0.44 ^d^ | 5.98 ± 2.86 |
| 6 | 4.39 ± 0.61^a^ | 1195 ± 207 | 8.39 ± 1.15 ^a^ | 2.27 ± 1.32 ^a,d,e^ | 0.51 ± 0.19 ^a,b,e^ | 1.7 ± 1.24 ^a,b,c^ |
| 12 | 4.91 ± 0.52 | 1110 ± 158 ^f^ | 9.52 ± 1.01 ^a^ | 1.89 ± 1.48 ^a,b,d,e^ | 0.22 ± 0.10 ^a,,b,c,d,e,f,g^ | 1.64 ± 1.42 ^b,c^ |
| 24 | 4.99 ± 0.95 | 539 ± 264 ^a^ | 9.33 ± 1.45 ^a^ | 1.85 ± 0.81 ^a,d,e^ | 0.42 ± 0.15 ^a,b,e^ | 1.35 ± 0.93 ^b,c^ |

3 week-old mice were subjected to trauma and hemorrhage (-48h) followed by mild (23G) polymicrobial cecal ligation and puncture (CLP) sepsis at 0h. At -48h, -42h, -36h, -24h, 0h, 6h, 12h and 24h, mice (6 per time point) were sacrificed and blood was collected and analyzed. RBC, red blood cell count; PLT, platelet count; Hb, hemoglobin; WBC, white blood cell count; NEU, neutrophil granulocyte, LYM, lymphocyte count.

^a^P<0.05 versus -48h, ^b^P<0.05 versus -42h, ^c^P<0.05 versus -36h, ^d^P<0.05 versus ‑24h, ^e^P<0.05 versus 0h, ^f^P<0.05 versus 6h, ^g^P<0.05 versus 24h, 05 versus all other time points. Data as mean ± SD; n ≥ 5 per time point.
