# Supplementary material for: A Non-Lethal Traumatic/Hemorrhagic Insult Strongly Modulates the Compartment-Specific PAI-1 Response in the Subsequent Polymicrobial Sepsis
Source: PLoS One. 2013 Feb 8;8(2):e55467. doi: 10.1371/journal.pone.0055467 (PMC3568129; doi:10.1371/journal.pone.0055467)
Supplement: Table S3 — Predictive accuracy of plasma PAI-1 for death. AUC, area under the curve; ROC, receiver operating characteristic; CI, confidence interval. *P<0.05. Plasma PAI-1 levels obtained were evaluated by the ROC curve to determine the predictive accuracy for the outcome as expressed by the AUC. The predictive accuracy of the ROC-AUC was defined as: 0.9–1 = excellent, 0.8–0.9 = good, 0.7–0.8 = fair, 0.6–0.7 = poor and <0.6 = not useful. (DOC) [file pone.0055467.s004.doc]

Table S3. Predictive accuracy of plasma PAI-1 for death.

| Time | AUC (ROC) | 95% CI | P-value |
| --- | --- | --- | --- |
| -48h | 0.56 | 0.35-0.76 | 0.584 |
| -24h | 0.63 | 0.46-0.81 | 0.153 |
| 0h | 0.62 | 0.44-0.79 | 0.200 |
| 6h | 0.57 | 0.38-0.75 | 0.475 |
| 24h | 0.65 | 0.47-0.83 | 0.110 |
| 48h | 0.81* | 0.65-0.98 | 0.006 |
| 72h | 0.93* | 0.82-1.04 | 0.001 |

AUC, area under the curve; ROC, receiver operating characteristic; CI, confidence interval

*P<0.05

Plasma PAI-1 levels obtained were evaluated by the ROC curve to determine the predictive accuracy for the outcome as expressed by the AUC. The predictive accuracy of the ROC-AUC was defined as: 0.9-1=excellent, 0.8-0.9=good, 0.7-0.8=fair, 0.6-0.7=poor and <0.6=not useful.
